# Conservative Treatment of Spontaneous Intraperitoneal Hemorrhage in Severe Dengue: A Case Report and Review

**DOI:** 10.4269/ajtmh.25-0296

**Published:** 2025-10-07

**Authors:** Panita Looareesuwan, Chaisith Sivakorn, Viravarn Luvira

**Affiliations:** ^1^ Department of Social and Environmental Medicine, Faculty of Tropical Medicine, Mahidol University, Bangkok, Thailand;; ^2^ Pulmonary Medicine and Pulmonary Critical Care, Bangkok Hospital, Bangkok, Thailand;; ^3^ Department of Clinical Tropical Medicine, Faculty of Tropical Medicine, Mahidol University, Bangkok, Thailand

## Abstract

Dengue fever is a common arboviral illness that can result in severe hemorrhagic complications, although spontaneous intraperitoneal bleeding remains rare. We report the case of a 26-year-old previously healthy female who presented with a 4-day history of high-grade fever and myalgia that was later diagnosed as dengue fever. During hospitalization, she developed vaginal bleeding followed by generalized abdominal pain and shock. Contrast-enhanced computed tomography revealed significant hemoperitoneum, despite a normal coagulation profile and no identifiable bleeding source. She was managed conservatively with supportive care and made a full recovery. A 1-year follow-up was unremarkable. This case underscores the importance of early recognition, prompt imaging, and consideration of differential diagnoses. In the absence of established guidelines for managing spontaneous intraperitoneal hemorrhage in dengue, individualized conservative treatment may be effective. Clinicians should remain vigilant for atypical presentations to ensure timely diagnosis and appropriate management.

## INTRODUCTION

Dengue fever, caused by dengue virus (DENV), is a mosquito-borne arboviral disease transmitted primarily by *Aedes aegypti*. It continues to pose a significant public health concern, with the global number of cases increasing steadily by 85.5% from 30.67 million in 1990 to 56.88 million in 2019.^1^ Clinically, dengue infection exhibits a wide spectrum of manifestations. Although the majority of symptomatic patients recover uneventfully with supportive care, fewer than 5% progress to severe and potentially life-threatening disease.^2^ According to the 2009 WHO Dengue Classification Scheme by dengue, severe dengue is classified by dengue, dengue with warning signs, and severe dengue.^3^ The criteria of severe dengue include severe plasma leakage, severe hemorrhage, or severe organ impairment.^3^

Bleeding manifestations in dengue are common and typically include mucocutaneous symptoms, such as epistaxis, petechiae, ecchymosis, gum bleeding, and hypermenorrhea. In severe cases, however, internal hemorrhages, such as gastrointestinal bleeding or more rarely, intraperitoneal hemorrhages, may occur and contribute to clinical deterioration. Unlike external bleeding, which is more easily recognized, internal hemorrhages can be subtle and more difficult to detect clinically. Other rare hemorrhagic complications associated with dengue include retinal hemorrhage^4^ and intracranial hemorrhage.^5^^,^^6^

This report describes a rare case of spontaneous intraperitoneal hemorrhage in a young patient with dengue fever, highlighting the importance of early recognition, targeted imaging, and individualized management strategies in the absence of specific treatment guidelines.

## CASE PRESENTATION

A previously healthy 26-year-old woman presented with a 4-day history of fever, myalgia, anorexia, and mild vaginal bleeding (two pads per day) occurring outside her menstrual cycle. She reported no history of trauma, pregnancy risk, or bleeding diathesis, including epistaxis, umbilical stump bleeding, prolonged bleeding after tooth extraction, or previous blood transfusion. There was no family history of bleeding disorders and no history of dengue vaccinations.

On admission, her vital signs were stable: temperature of 37.6°C, respiratory rate of 16 breaths per minute, pulse of 92 beats per minute, and blood pressure of 105/57 mm Hg. Oxygen saturation was 98% on room air. Physical examination revealed mild epigastric tenderness without hepatosplenomegaly; no other abnormalities were noted. Dengue virus infection was confirmed by positive NS1 antigen and dengue IgM, with a negative dengue IgG. The dengue IgM/IgG ratio measured using the TECAN SUNRISE microplate reader (Tecan Group Ltd., Männedorf, Switzerland) was 2.16, exceeding the threshold of 1.78 and suggestive of primary dengue infection.^7^ Dengue polymerase chain reaction was positive for DENV1 (CFX96^™^ Real-Time PCR Detection System, Bio-Rad Laboratories, Hercules, CA).

On day 5 of illness, laboratory investigations revealed mild anemia (hemoglobin: 10.3 g/dL), leukopenia (1,000 cells/mm^3^), thrombocytopenia (46,000 cells/mm^3^), and mild transaminitis (aspartate aminotransferase: 108 IU/L, alanine aminotransferase: 38 IU/L). Chest radiography showed no evidence of pulmonary leakage. Because of concerns of potential clinical deterioration and further bleeding, the patient was admitted on day 5 of fever for close monitoring. Intravenous hydration with 5% dextrose normal saline solution at a rate of 40 mL/hour and norethisterone (Primolut N) was initiated.

**Figure 1.**
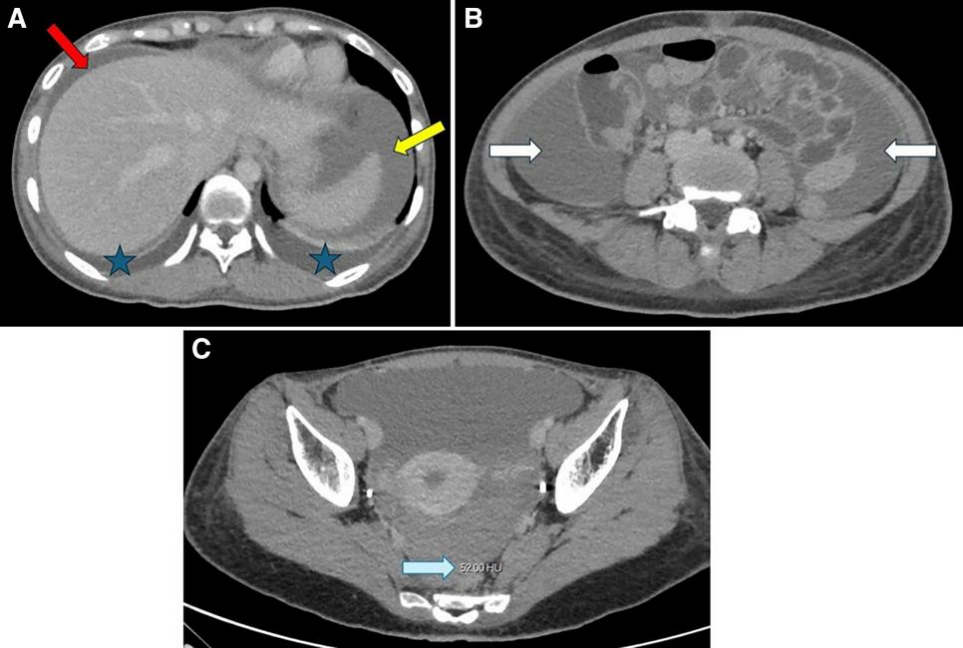
Contrast-enhanced computed tomography images show a large volume of hemoperitoneum in (**A**) the perihepatic region (red arrow), (**A**) the perisplenic region (orange arrow), and (**B**) the bilateral paracolic gutters (white arrows). (**A**) Bilateral pleural effusion is noted in both lungs (blue star). The liver and spleen appear normal. (**C**) The pelvic cavity has a region of interest measurement of 52 Hounsfield units (HU; blue arrow).

On day 8, the patient developed generalized abdominal pain without peritoneal signs along with hypotension, tachycardia, and poor capillary refill, consistent with severe dengue. Repeat laboratory tests demonstrated worsening anemia (hemoglobin: 7.5 g/dL), thrombocytopenia (20,000 cells/mm^3^), normal coagulation parameters, worsening transaminitis, and hypocalcemia. Bedside ultrasound revealed intraperitoneal free fluid in the cul-de-sac, splenorenal, and hepatorenal spaces, with hyperechoic density suggestive of intraperitoneal bleeding. Her urine pregnancy test was negative. After initial stabilization, contrast-enhanced computed tomography (CT) of the abdomen was performed, which showed that the liver, spleen, both ovaries, and blood vessels were unremarkable. There was no evidence of active extravasation (Figure 1). Factor V Leiden, Von Willebrand factor, and protein C were normal, with a slight decrease in protein S activities. She was transfused with a total of 6 units of leukocyte-poor red cells, 6 units of leukocyte-poor platelet concentrate, 1 unit of fresh-frozen plasma, and 10 units of cryoprecipitate, and her vital signs then stabilized. The patient was discharged after 10 days of inpatient stay.

One year after discharge, she remained well without the need for surgical intervention. She was followed up three times, with no recurrence of hemorrhagic symptoms. The laboratory values during hospitalization and follow-up are summarized in Table 1.

**Table 1:** Summary of laboratory findings during admission

Year	2021										2022	Reference Values
Dates	December 25	December 26	December 27	December 28	December 29	December 30	January 1	January 3	January 13	February 10	February 9	
Blood transfusion	–	–	–	4 LPRC, 2 LPPC, 10 Cryo, 1 FFP	2 LPRC, 1 LPPC	3 LPPC	–	–	–	–	–	–
Day of fever	5	6	7	8*	9	10	12	14	10 days after discharge	38 days after discharge	1 year after discharge	–
Hematocrit (%)	31.6	30	34	22.1	37	34	39	41	48.5	37.5	37	37–47
Hemoglobin (g/dL)	10.3	10	11	7.5	12	11	13	14	16	12.7	12	12–16
White blood cells (10^3^/*µ*L)	1,000	800	900	1,600	4,700	6,500	6,100	4,800	5,800	5,400	4,500	5–10
Neutrophil (%)	60	45	21	46	66	75	33	44	53.7	58.2	69.7	45–74
Lymphocyte (%)	21	35	54	29	17	15	45	35	36.2	32	22.5	16–45
Atypical lymphocyte (%)	13	10	13	16	9	3	7	2	–	–	–	0–5
Absolute neutrophil count (%)	600	360	189	736	3,102	4,875	2,013	2,112	5,800	3,143	3,137	–
Platelet count (per *µ*L)	46,000	36,000	25,000	20,000	142,000	161,000	284,000	395,000	346,000	237,000	260,000	150,000–450,000
Sodium (mmol/L)	138	–	–	133	135	136	–	140	140	140	140	136–145
Potassium (mmol/L)	4	–	–	4	3.7	3.4	–	5	4.4	4.4	4.2	3.5–5.1
Chloride (mmol/L)	109	–	–	105	109	107	–	107	103	103	105	98–107
Bicarbonate (mmol/L)	22	–	–	19	16	18	–	22	25	25	–	22–29
Calcium (mg/dL)	–	–	–	7.2	–	–	–	–	–	–	9.8	8.6–10
Lactate (mg/dL)	–	–	–	10.6	7	7.9	–	–	–	–	–	4.5–19.8
BUN (mg/dL)	6.1	–	–	5.8	5.5	4.5	–	9.1	9.3	6.8	8.5	6–20
Creatinine (mg/dL)	0.57	–	–	0.63	0.52	0.4	–	0.49	0.6	0.61	0.66	0.51–0.95
Aspartate aminotransferase (U/L)	108	–	–	610	356	206	135	98	56	38	14	0–32
Alanine aminotransferase (U/L)	38	–	–	335	223	182	141	122	77	47	19	0–33
Alkaline phosphatase (U/L)	39	–	–	42	38	41	–	51	91	71	68	35–104
Albumin (g/dL)	3.7	–	–	3	2.8	3	–	3.5	5	4.5	5.3	3.5–5.2
Globulin (g/dL)	2.2	–	–	2	1.9	3	–	2.7	3.4	2.7	2.3	2.5–3.5
Total protein (g/dL)	5.9	–	–	5	4.7	5.3	–	6.2	8.4	7.2	7.5	6.6–8.7
Total bilirubin (mg/dL)	0.2	–	–	0.2	0.4	0.4	–	0.7	1	0.5	0.3	0.0–1.2
Direct bilirubin (mg/dL)	0.1	–	–	0.2	0.2	0.3	–	0.2	0.3	0.2	0.2	0.0–0.3
PT/PTT	10/26	–	–	10/22	10/22	11/20	11/20	11/20	11/25.77	–	–	10.3–12.3/20.8–28.4
TT	–	–	–	–	–	14.5	–	–	16.5	–	–	15.8–22.2
INR	0.92	–	–	0.98	0.96	1	1.05	1.04	0.97	–	–	–
Fibrinogen	–	–	–	206	–	–	–	–	–	–	–	179–398
Others	Day 5: NS1 antigen and dengue IgM positive, dengue IgG negative; factor V Leiden: negative; Von Willebrand factor (ristocetin cofactor activities): 107 (reference: 50–150); protein C: 81 (reference: 70–140); protein S: 26 (reference: 59–118); dengue serotype 1 (Ct: 14.46)											

BUN = blood urea nitrogen; Cryo = cryoprecipitate; Ct = cycle threshold; FFP = fresh-frozen plasma; INR = international normalized ratio; LPPC = leukocyte-poor platelet concentrate; LPRC = leukocyte-poor red cells; PT = prothrombin time; PTT = partial thromboplastin time; TT = thrombin time.

* Intraperitoneal bleeding.

## DISCUSSION

The final diagnosis in this case is severe dengue as the patient fulfilled WHO dengue classification (2009) criteria for severe hemorrhage. Dengue-associated intra-abdominal hemorrhage is a rare but potentially life-threatening complication. From our review, 16 cases of dengue-associated intraperitoneal bleeding have been reported, with splenic rupture and hematoma as the most common causes (11 of 16 cases) followed by corpus luteal cyst rupture (4 cases) and spontaneous bleeding without identified cause (1 case) as shown in Table 2.^8^^–^^21^

**Table 2:** Published cases of intraperitoneal bleeding associated with dengue infection

No.	Authors (year)	Patient Profile	Dengue Diagnosis	Days of Bleeding	Presenting Symptoms of IH	Blood Investigation during IH	Paracentesis or Imaging Modality Used	Site of Bleeding	Treatment	Outcome
1	Redondo et al.^8^ (1997)	23-year-old female, healthy	Dengue IgM positive	5	Hypovolemic shock and severe diffuse abdominal pain	Hb 6.5 Hct 18.3 WBC 13,000 N 40 L 50 Plt 42,000 AST 157 ALT 54 LDH 1,204 Prolonged PT and APTT Decreased fibrinogen	USG abdomen: abdominal bleeding	Splenic rupture	Laparoscopic exploration twice, splenectomy	Died, sepsis and multiorgan dysfunction
2	Miranda et al.^9^ (2003)	52-year-old woman, U/D DM, HT, anxiety	Dengue IgM positive	6	Sudden severe abdominal pain	Hct 25.1 WBC 3,000 N 41 L 52 Band 1 Plt 67,000 AST 228 ALT 126	CTWA: large splenic hemorrhage	Splenic rupture	Laparoscopic exploration, splenectomy	Survived
3	Seravali et al.^10^ (2008)	27 year old	Dengue IgM and IgG positive	NA	Generalized abdominal pain	NA	Peritoneal lavage positive for blood	Splenic hematoma	Laparoscopic exploration (3L), splenectomy	Survived
4	Seravali et al.^10^ (2008)	20-year-old male	Dengue IgM and IgG positive	4	Severe abdominal pain and low arterial blood pressure	WBC 45,300 Plt 46,000 AST 3,964 ALT 1,240	CTWA: subcapsular spleen hematoma	Splenic hematoma	Laparoscopic exploration, splenectomy	Survived
5	de Moura Mendonça et al.^11^ (2011)	33-year-old male	Dengue NS1 antigen and IgM positive	4	Blumberg sign positive, hypotension, tonic–clonic convulsion, and hypoxemia	Hct 49.8 WBC 3,100 Plt 27,000 ALT 404 GGT 223 Alb 2.5 (initial laboratory)	Autopsy revealed splenic rupture	Splenic rupture	Conservative treatment	Died
6	Bhaskar and Moorthy^12^ (2012)	26-year-old male, healthy	Dengue NS1 antigen positive, dengue IgM and IgG negative (day 5), dengue IgM and IgG positive (day 8)	5	Soft abdomen with diffuse tenderness	Hb 12.5 WBC 8,000 Plt 40,000 BUN 28 Cr 1.2 AST 80 ALT 60 ALP 130 INR 1.2 PT 36	USG abdomen: possible hematoma or abscess in the spleen CTWA: splenic rupture with intra- and perisplenic hematoma	Splenic rupture	Surgery with splenectomy	Survived
7	Chandrashekar et al.^20^ (2013)	43-year-old male, U/D DM	Dengue IgM:IgG = 4:1 (initial), dengue IgM:IgG = 5:2 (14 days later)	NA	Abdominal distension and pain	Hb 6.9 Hct 23 PT 15.9 APTT 29 INR 1.84	Paracentesis: 500 mL hemorrhagic ascites, USG	Spontaneous bleeding	Laparoscopic exploration (1.5 L of peritoneal fluid and blood)	Survived
8	Mukhopadhyay et al.^13^ (2014)	26-year-old female	Dengue NS1 antigen and IgM positive	4–5	Hypotension, tachycardia, and diffuse abdominal pain	Hb 3.4, WBC 3,300 N 90 L 8 E 2 Plt 40,000 BUN 88 Cr 2	Paracentesis: hemoperitoneum CTWA: perisplenic hypoattenuating collection	Splenic rupture	Conservative treatment	Survived
9	De Silva and Gunasekera^14^ (2015)	28-year-old man, healthy	Dengue IgM and IgG positive	8	Hypotension, tachycardia, abdominal distension, and severe diffuse abdominal pain	Hb 8.8 WBC 4,000 Plt 90,000 Normal coagulation profile	CTWA: free fluid in the peritoneal cavity with a perisplenic hematoma	Splenic rupture	Laparoscopic exploration showed splenic laceration (4 L of blood), splenectomy	Survived
10	Padyana et al.^15^ (2020)	28-year-old male	Dengue NS1 antigen positive	5	Hypotension, tachycardia, and diffuse abdominal tenderness without guarding	Hb 11 WBC 21,690 Plt 32,000 PT 14.6 PTT 53.2 INR 1.2 AST 118 ALT 52 Lipase 60	USG abdomen: gross ascites Paracentesis: hemorrhagic fluid CTWA: hemoperitoneum	Splenic hematoma with venous extraversion	Embolization of splenic artery	Survived
11	Ungthammakhun et al.^16^ (2021)	22-year-old male, HbHCs, G6PD	Dengue NS1 antigen positive, RT-PCR DEN1	2	Hypotension and severe left-upper-quadrant abdominal pain	Hb 6.2 WBC 18,500 N 87 L 5 Plt 111,000	CT upper abdomen: ruptured spleen and subcapsular hematoma	Splenic rupture and hematoma	Laparoscopic exploration, splenectomy	Survived
12	Puligunta^17^ (2022)	27-year-old male	Dengue NS1 antigen positive	5	Hypotension, tachycardia, and severe diffuse abdominal pain	Hb 5.1 Plt 30,000	CTWA: splenic rupture	Splenic rupture	NA	NA
13	Pahari et al.^18^ (2023)	54-year-old male, healthy	Dengue NS1 antigen and IgM positive	10	Marked tender at left hypochondrium	Hb 12.3 WBC 8,300 N 79 L 22 Plt 60,000	USG abdomen: splenic hematoma CTWA: grade II splenic hematoma	Splenic rupture	Conservative treatment	Died, septic shock and hospital-acquired pneumonia
14	Raajput et al.^21^ (2020)	21-year-old female	Dengue NS1 antigen positive, dengue IgM and IgG negative	3	Mild tenderness in lower abdomen	Hb 8.8 WBC 8,900 Plt 15,000 PT 17 APTT 27 INR 1.18	USG abdomen: 500 mL free fluid in peritoneal cavity with internal echoes Paracentesis: hemorrhagic fluid	Corpus luteal cyst rupture	Laparoscopic exploration, left salpingo-oophorectomy	Survived
15	Kulshrestha et al.^19^ (2024)	21-year-old female	Dengue NS1, IgM, and IgG positive	4	Tenderness in left iliac fossa	Hb 4.8 WBC 7,400 Plt 160,000 PT 47 INR 2.1	USG abdomen: left fallopian tube edematous	Corpus luteal cyst rupture	Laparoscopic exploration (1.5 L of hemoperitoneum), cystectomy	Survived
16	Kulshrestha et al.^19^ (2024)	35-year-old female	Dengue NS1 antigen, IgM, and IgG positive	NA	Abdominal distension and tenderness at lower abdomen	Hb 3.4 WBC 2,415 Plt 115,000 BUN 30.6 Cr 1.7 AST 370 ALT 387 PT 45 INR 1.18	Paracentesis: hemorrhagic fluid USG abdomen: anechoic left ovarian cyst with massive free fluid	Corpus luteal cyst rupture	Laparoscopic exploration (2 L of hemoperitoneum), peritoneal lavage	Survived
17	Kulshrestha et al.^19^ (2024)	33-year-old female	Dengue NS1, IgM, and IgG positive	NA	Hypotension, tachycardia and left lower abdominal pain	Hb 8.5 WBC 18,000 Plt 106,000 BUN 25.6 Cr 1.2 AST 304 ALT 220 INR 1.18	USG: anechoic left ovarian cyst with massive hemoperitoneum	Corpus luteal cyst rupture	Laparoscopic exploration, cystectomy	Survived
18	This case	26-year-old female	PCR DEN1 positive, NS1 antigen and dengue IgM positive, dengue IgG negative	8	4 days of fever, myalgia, anorexia, and mild vaginal bleeding	Hb 7.5 Hct 22.1 WBC 4,700 N 46 L 29 Plt 20,000 AST 610 ALT 335 Normal PT/PTT	CTWA: large amount of hemoperitoneum Normal liver, spleen, and ovaries	Spontaneous bleeding	Conservative	Survived

Alb = albumin; ALP = alkaline phosphatase; ALT = alanine aminotransferase (units per liter); APTT = activated thromboplastin time; AST = aspartate aminotransferase (units per liter); BUN = blood urea nitrogen (milligrams per deciliter); Cr = creatinine (milligrams per deciliter); CT = computed tomography; CTWA = contrast-enhanced computed tomography of whole abdomen; DM = diabetes; E = eosinophils; G6PD = glucose-6-phosphate dehydrogenase deficiency; GGT = gamma-glutamyl transferase; Hb = hemoglobin (grams per deciliter); HbHC = hemoglobin H constant spring disease; Hct = hematocrit (percentage); HT = hypertention; IH = intraperitoneal hemorrhage; INR = international normalized ratio; L = lymphocytes; LDH = lactate dehydrogenase; N = neutrophils; NA = not applicable; PCR = polymerase chain reaction; Plt = platelet; PT = prothrombin time; PTT = partial thromboplastin time; RT-PCR = reverse transcription polymerase chain reaction; U/D = underlying; USG = ultrasound; WBC = white blood cells (per millimeter cubed). Direct bilirubin is in units of milligrams per deciliter. Platelets are per millimeter cubed. Total bilirubin is in units of milligrams per deciliter.

To date, this represents the second reported case of spontaneous intraperitoneal hemorrhage in a dengue patient. The patient, a previously healthy 26-year-old woman, developed intra-abdominal bleeding on day 8 after fever onset. Laboratory investigations revealed no underlying bleeding disorders or coagulopathies.

This review demonstrates patients ranging in age from 20 to 54 years old with bleeding occurring 2–10 days after fever onset, most frequently around days 4 and 5, coinciding with the critical plasma leakage phase of dengue infection. Generalized abdominal pain and distension were the predominant symptoms, with nine cases presenting with hypovolemic shock and one case presenting with convulsions. Hemoglobin and hematocrit levels were often low, consistent with active hemorrhage, and thrombocytopenia was observed in all cases (mostly <100,000/mm^3^). Ultrasound and CT scans of the abdomen were the primary imaging modalities, where the latter provided essential information on the extent and source of bleeding. In selected cases, invasive procedures, such as paracentesis, were performed to confirm hemoperitoneum. The primary sites of dengue-associated intraperitoneal hemorrhage include splenic rupture or hematoma,^8^^–^^18^ corpus luteal cyst rupture,^19^ and spontaneous bleeding of unknown origin.^20^

Nontraumatic splenic rupture is a rare entity, with etiologies including neoplasm (30.3%), infections (27.3%), inflammatory and noninfections (20%), drug- and treatment-related causes (9.2%), mechanical disorder (6.8%), and cases involving normal spleen (6.4%).^22^ Among infectious causes, the most frequently implicated pathogens include the Epstein–Barr virus (infectious mononucleosis), cytomegalovirus, bacterial endocarditis, and malaria.^22^ Clinically, nontraumatic splenic rupture presents with sudden left-upper-quadrant pain, abdominal rigidity, and referred pain to left shoulder because of diaphragmatic irritation (Kehr’s sign).^23^ The pathophysiology of splenic rupture in dengue remains incompletely understood, although proposed mechanisms include a combination of splenic congestion, thrombocytopenia, and coagulopathy that may lead to splenic rupture. Management strategies vary; a review by Renzulli et al.^22^ reported that 84.1% of nontraumatic splenic ruptures underwent total splenectomy, whereas 14.7% were managed conservatively, and 1.2% were treated with organ-preserving surgery.^23^ The treatment choice depends on hemodynamic stability of the patient and etiology of rupture.^24^ The nontraumatic splenic rupture-related mortality rate was 12.2%.^22^

Corpus luteal cyst rupture has been reported in four cases of dengue-associated intraperitoneal hemorrhages. As its clinical presentation may mimic ectopic pregnancy or other pregnancy-related complications, pregnancy should be systematically excluded in reproductive-age women presenting with abdominal pain and a drop in hematocrit. A pregnancy test should be performed as part of the differential diagnosis workup as failure to distinguish between dengue-associated hemorrhage and ectopic pregnancy may lead to delays in appropriate management, with potential implications for patient outcomes. Management of corpus luteal cyst rupture is guided by hemodynamic stability and the extent of hemoperitoneum, with options ranging from conservative management to exploratory laparoscopy or laparotomy. Current trends favor laparoscopy and conservative treatment over laparotomy, reflecting a shift toward minimally invasive approaches.^25^ In this case, the patient tested negative for urine pregnancy test, ruling out a pregnancy-related cause of hemorrhage.

Diagnosis of intraperitoneal hemorrhage in dengue cases requires clinical suspicion and imaging. Although paracentesis is a rapid procedure that can confirm hemorrhagic fluid, it is invasive and does not provide information on the bleeding source, necessitating further imaging in most cases. Contrast-enhanced computed tomography of whole abdomen (CTWA) is considered the gold standard for diagnosing spontaneous intraperitoneal bleeding, offering detailed anatomical visualization of the peritoneal cavity, identifying hemorrhagic sources, and detecting active extravasation or vascular abnormalities that may warrant embolization. A Hounsfield unit of more than 30 in free fluid strongly suggests hemoperitoneum, except in cases of bleeding more than 48 hours old, whereas lower values may indicate nonhemorrhagic ascites.^26^

## CONCLUSION

Despite dengue being a well-recognized cause of coagulopathy and thrombocytopenia-related hemorrhage, there are no established guidelines for managing spontaneous intraperitoneal bleeding in dengue patients. In this case, hemoperitoneum was diagnosed using bedside ultrasound and CTWA without paracentesis. The patient was managed conservatively with aggressive resuscitation and extensive clinical monitoring, achieving full recovery. Although dengue hemorrhagic fever is typically associated with secondary infections, severe manifestations can also occur in the context of primary infection as demonstrated in this case. By contrast, a previously reported case of spontaneous hemoperitoneum in a 43-year-old man required confirmation through abdominal paracentesis and subsequent laparoscopic exploration, leading to recovery.^20^ Thus, this case highlights that the management decision is determined on a case-by-case basis. Early recognition and vigilant clinical monitoring remain critical, and conservative management may be a feasible option in selected patients.
